# Process Window for Highly Efficient Laser-Based Powder Bed Fusion of AlSi10Mg with Reduced Pore Formation

**DOI:** 10.3390/ma14185255

**Published:** 2021-09-13

**Authors:** Artur Leis, Rudolf Weber, Thomas Graf

**Affiliations:** 1 Institut für Strahlwerkzeuge (IFSW), University of Stuttgart, Pfaffenwaldring 43, 70569 Stuttgart, Germany; rudolf.weber@ifsw.uni-stuttgart.de (R.W.); thomas.graf@ifsw.uni-stuttgart.de (T.G.); 2 Graduate School of Excellence Advanced Manufacturing Engineering (GSaME), University of Stuttgart, Nobelstraße 12, 70569 Stuttgart, Germany

**Keywords:** LPBF, additive manufacturing, process efficiency, pores, keyhole, AlSi10Mg

## Abstract

The process window for highly efficient laser-based powder bed fusion (LPBF), ensuring the production of parts with low porosity, was determined by analyzing cross-sections of samples that were generated with laser powers varying between 10.8 W and 1754 W, laser beam diameters varying between 35 μm and 200 μm, and velocities of the moving laser beam ranging between 0.7 m/s and 1.3 m/s. With these parameters, the process alters between different modes that are referred to as simple heating, heat conduction melting (HCM), key-bowl melting (KBM), and deep-penetration melting (DPM). It was found that the optimum process window for a highly efficient LPBF process, generating AlSi10Mg parts with low porosity, is determined by the ratio *P_L_*/*d_b_* of the incident laser power *P_L_* and the beam diameter *d_b_* of the beam on the surface of the bead, and ranges between *P_L_*/*d_b_* = 2000 W/mm and *P_L_*/*d_b_* = 5200 W/mm, showing process efficiencies of about 7–8%. This optimum process window is centered around the range *P_L_*/*d_b_* = 3000–3500 W/mm, in which the process is characterized by KBM, which is an intermediate process mode between HCM and DPM. Processes with *P_L_*/*d_b_* < 2000 W/mm partially failed, and lead to balling and a lack of fusion, whereas processes with *P_L_*/*d_b_* > 5200 W/mm showed a process efficiency below 5% and pore ratios exceeding 10%.

## 1. Introduction

Laser-based powder bed fusion (LPBF) is characterized by the fusion of multiple melt beads in consecutively stacked layers, which allows the generation of highly complex parts. The process parameters that are required to achieve high build rates during the process and low porosity in the manufactured parts, which are both fundamental for LPBF, are typically determined experimentally.

Current developments scope the increase in the build rate by parallelizing the LPBF process with multiple laser beams or to decrease the non-productive times by improving the concepts of the LPBF machines [1]. Matilainen et al. investigated the influence of the process parameter on the cross-sectional dimensions of the generated melt beads as a function of the energy density in stainless steel. They applied 200 W and 325 W of laser power, and varied the scanning speed between 400 mm/s and 2600 mm/s at otherwise constant parameters, and it was found that the penetration depth and the cross-sectional area of the melt bead increase with increasing energy density, whereas the width-to-depth ratio decreases with increasing energy density, due to the formation of a keyhole [2]. Hyer et al. reported on a wide parameter study of AlSi10Mg, and investigated the relative density, hardness, cell size, and melt pool dimensions as functions of the incident laser power, the scanning speed, and the energy density, and presented similar behavior of the melt pool for AlSi10Mg, as shown by Matilainen et al. [3]. Mishra et al. investigated the influence of the hatch distance on the process efficiency at otherwise identical process parameters, and derived an equation to calculate the process efficiency. They found that the process efficiency of LPBF can be calculated to be 2–20% when the hatch distance is varied [4]. Tenbrock et al. analyzed the present melting modes at LPBF, according to the intensity distribution of the laser beam, and found that for a top-hat shaped intensity distribution, the melting modes heat conduction melting and melting with the formation of a keyhole can be described by a function of the line energy and the intensity of the laser beam on the surface of the workpiece [5]. From this, it was concluded that the process efficiency is limited by an intensity threshold and a critical energy density of the applied material. Patel et al. developed quantities of the dimensionless heat input as a function of the dimensionless scanning speed, to predict the melting modes and their transitions, which are present at LPBF. Since no melt pools of the melting with the formation of a keyhole were reported for AlSi10Mg, no prediction of the keyhole was made [6]. However, although a large number of investigations on the melt pool sizes and the melting modes have already been carried out, the influence of the process parameters on the process efficiency and the formation of pores is not yet fully understood.

The process efficiency is defined as the ratio between the power that is required to melt a specific volume per time and the incident laser power [4,7]. For LPBF, this specific volume solely results from the melting of the amount of material that is added to a bead [4]. To some extent, the physical process may be compared to laser beam welding [8], where the material is either melted (heat-conduction welding) or partially evaporated to generate a keyhole (deep-penetration welding) [9], as vividly summarized by Oliveira et al. [10]. In particular, remote laser beam welding with a scanner, which is widely used for welding tasks in automotive production [11] or for the welding of thin foils [12,13], is very similar to LPBF. As is known from laser beam welding, the process efficiency is strongly influenced by the process parameter and the resulting process mode. Compared to heat-conduction welding, deep-penetration welding exhibits a significantly higher process efficiency, due to the increased absorptance as a consequence of the multiple reflections of the laser radiation within the keyhole [7,14,15]. However, such an increase in the process efficiency applies solely to laser beam welding processes, whose process efficiency is derived from the volume of the total molten material, while for LPBF solely, the amount of the material that is added to the bead contributes to the process efficiency.

In laser beam welding, a further increase in the process efficiency can be achieved by increasing the process velocity, also called scanning speed for LPBF because of the scanned operation, in order to reduce losses due to heat conduction [4,16]. However, this effect saturates for high process velocities exceeding about 0.2 m/s [17]. This results in a negligible small influence of the scanning speed on the heat losses within the range of the applied scanning speeds of LPBF processes.

The formation of pores tends to be even more sensitive to changes in the process parameters in LPBF processes. In order to analyze and to reduce the porosity of parts manufactured by LPBF, various parameter studies have already been presented [18,19,20,21,22]. It was shown that the main reasons for the formation of pores during LPBF may, on the one hand, be a non-steady formation of the melt pool, also known as ‘balling’, which results in non-continuous beads and a lack of fusion between the single layers [23]. On the other hand, pores originate from instabilities of the keyhole, when the LPBF process is running in the deep-penetration melting mode [9,24,25,26,27,28].

The consideration of both the process efficiency and the formation of pores is essential to define suitable process parameters, and to identify the process limits of LPBF. Therefore, the present paper reports on the determination of the process window for single-beam LPBF, with the goal to identify the parameters that lead to a high process efficiency and a low porosity of the produced parts within the single melt bead. To this end, the experiments covered different process parameters, resulting in different process modes, which we termed heating (when the material is not melted), heat conduction melting (HCM), deep-penetration melting with the formation of a keyhole (DPM), and the transition between HCM and DPM, here called key-bowl melting (KBM) (also referred to as transition mode melting or vapor depression melting) in analogy to the terms used in laser beam welding [13,29]. The modes mainly differ with respect to the attained maximum temperature, which, for a given material and the comparably low applied scanning speed *v* (i.e., small Péclet numbers $Pe=\left(d_{b}\cdot v\right)/\kappa ≲16/\pi$, where $d_{b}$ is the diameter of the laser beam on the surface of the workpiece, v is the scanning speed, $\kappa =\lambda_{th}/\left(\rho \cdot c_{P}\right)$ is the temperature conductivity, $\lambda_{th}$ is the thermal conductivity, ρ is the density, and $c_{P}$ is the specific heat capacity of the material), is known to essentially depend on the ratio $P_{L}/d_{b}$ of the incident laser power $P_{L}$ and the beam diameter $d_{b}$ on the surface of the workpiece [7,30,31]. Therefore, the experiments were set up to a maximum Péclet number of 4.23, so that the influence of the scanning speed is negligible. Reducing the possible process parameters to the incident laser power $P_{L}$ and the beam diameter $d_{b}$ on the surface of the workpiece, and considering the process modes that depend on them, can contribute significantly to understand the LPBF process. In this study, AlSi10Mg was used, which impresses with its mechanical properties combined with its good processability for LPBF, which has already been investigated in a number of studies [3,18,21,22,32,33].

In the following, the experimental setup, and the generation, preparation, and analysis of the samples are described first. This is followed by the investigation of the influence that the ratio $P_{L}/d_{b}$ has on the process efficiency and the porosity. The discussion of the results finally leads to the optimum process window.

## 2. Materials and Methods

For LPBF the process efficiency may be expressed by the following [4,7]:

(1) $$\eta_{P}=\frac{A_{a}\cdot v\cdot \rho \cdot \left[c_{P}\cdot \left(T_{m}-T_{0}\right)+h_{s}\right]}{P_{L}},$$

where $A_{a}$ is the fraction of the cross-sectional area of the generated bead that is attributed to the material, which is freshly added (hence the molten and solidified powder; see below) to the bead, v is the traverse speed of the scanned laser beam, ρ is the density of the solid material in the bead, $c_{P}$ is the mass-specific heat capacity of the solid material, *T_m_* the melting temperature of the processed material, *T*_0_ the ambient temperature, *h_s_* the mass-specific melting enthalpy of the processed material, and *P_L_* is the incident laser power.

**Figure 1:**
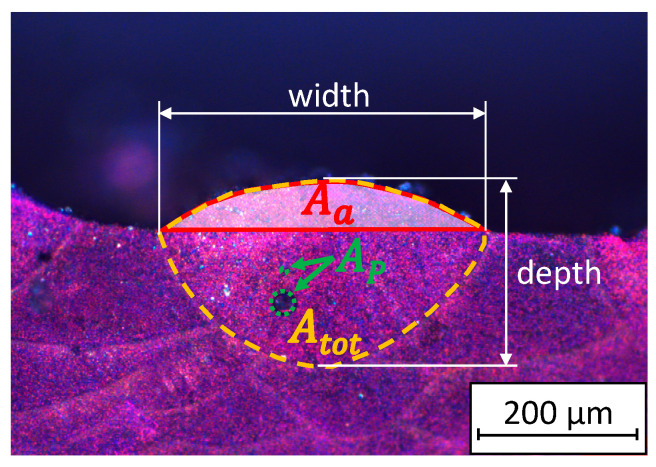
Cross-section of a processed sample, which was anodically etched according to Barker [34]. The total area of the melt bead $A_{tot}$ (orange dashed line) and the area $A_{a}$ of the added layer (red line and filling in white) were used to determine the process efficiency. The cross-sections of the pores $A_{P}$ that are contained within the analyzed bead are marked by green dashed lines.

When the process efficiency is experimentally determined from cross-sections of the generated melt bead as shown in Figure 1, one must consider that $A_{a}\cdot v\cdot \rho$ is the time-specific mass of the processed material that is added to the processed seam. As the bead generated by LPBF is formed from both the newly added material and from remelted material of the previous layer, the effective area $A_{a}$ is smaller than the total cross-sectional area of the bead. It follows that the process efficiency equals 0% if no material of the powder layer is metallurgically fused to the substrate (i.e., $A_{a}=0$ μm^2^). The process efficiency equals 100% if the incident laser power is completely absorbed (i.e., absorptance = 100%) by the powder and is directly fused to the substrate. However, the substrate may not be melted (i.e., $A_{a}$ = $A_{tot}$) and no energy losses are considered. Thus, in reality, the process efficiency of 100% is not achievable. The surface of the substrate at the sides of the melt bead was used as a reference to distinguish between the original layer and the added material in the new melt bead.

The cross-sections were also used to quantify the porosity of the generated samples. The layer-specific pore ratio was calculated by $A_{P}/A_{a}$, where $A_{P}$ is the cross-sectional area of pores within the total area of the melt bead $A_{tot}$, as illustrated in Figure 1 with green dashed lines and with orange dashed lines, respectively. Hence $A_{P}/A_{a}$ relates the pores left in the freshly generated bead to the new material added to the bead.

In order to investigate the influence of the process parameters on the process efficiency $\eta_{P}$ and on the formation of pores, melt beads were generated by applying a wide range of different processing parameters and analyzed as sketched above.

As the envisaged wide parameter field cannot be realized on conventional machines, the approach was to produce additively manufactured samples with a TruPrint3000 LPBF machine (with 450 W of maximum laser power and minimum beam diameter of 100 μm) to produce as realistic conditions as possible and afterwards to perform an additional fusion process of an additional powder layer on top of these pre-manufactured samples in a separate experimental setup, which provided wider ranges of available power, scanning speed and beam diameters.

**Figure 2:**
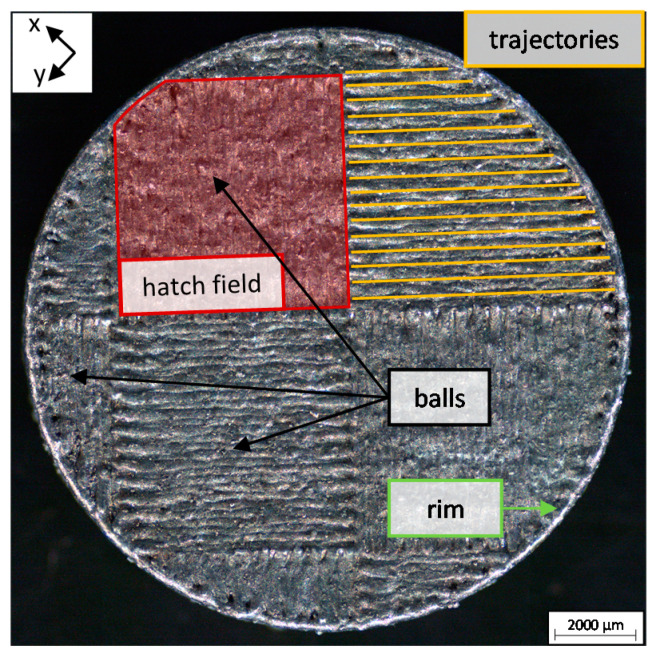
Top view of an additively manufactured sample (without re-melting of the last layers), which was used for the experiments.

The material used was AlSi10Mg-A LMF with a distribution of the diameter of the powder grains of 20 μm to 63 μm. The samples generated in the TruPrint3000 LPBF machine were produced using the standard parameter set, i.e., a laser power of $P_{L}$ = 430 W, a scanning speed of v = 1.3 m/s, and a beam diameter on the sample’s surface of $d_{b}$ = 100 μm. The generated samples were of cylindrical shape with a diameter of 15 mm and a height of 2 mm. A standardized chess pattern with perpendicularly oriented hatching in the fields measuring 5.89 mm by 5.89 mm and with a hatch distance of 210 μm was used for the LPBF processing, as shown in Figure 2. The chess pattern was shifted by 4.02 mm in the x-direction and by 5.44 mm in the y-direction at a 45° angle to the orientation of the hatching for each new layer. The hatch fields were automatically truncated at the circumference of the samples as observed in Figure 2.

The height of the powder layer was set nominally to 60 μm for the production of the samples. In order to ensure realistic LPBF process conditions in the subsequent investigations performed on these samples in the separate experimental setup, the commonly applied re-melting of the top layers at the end of the generation of the samples was omitted. The single trajectories of the hatch and the square hatched fields are highlighted in Figure 2 by the orange lines and the red area, respectively. Additionally, singular balls on the surface of the sample can be observed in the picture, which are a result of spattering. The roughness of the surface of the pre-manufactured samples was measured using a Keyence 3D laser scanning microscope (LSM) VK-9710-K and was to be $S_{a}=22.3{\;\mu m\;}_{-7.3\;\mu m}^{+4.7\;\mu m}$. This is the effective surface roughness, which applies to each single layer that is produced during the LPBF processing. The significantly increased roughness of the surface of the additively manufactured sample leads to a variation in the local absorptance and the local inhomogeneity of the applied powder layer during the LPBF process. A difference in the behavior of the absorptance can be observed for machined samples [35,36] and for additively pre-manufactured samples [37]. In order to consider the conditions, which are present during LPBF, and to determine the influence of the incident laser power $P_{L}$ and the beam diameter $d_{b}$ on the surface of the workpiece on the process efficiency, pore ratio, and aspect ratio, additively pre-manufactured samples were used instead of machined metal parts for the investigation.

The setup for the experimental investigations of the LPBF process, which provided significantly wider ranges of the processing parameters, was equipped with an SPI QUBE fiber laser with a diameter of the fiber core of 25 μm, a maximum laser power of 2000 W, and a wavelength of 1075 nm ± 2 nm [38]. A scanner (Scanlab intelliSCAN 30) with a collimating length of 200 mm and a focusing length of 340 mm was used to move the laser beam on top of the surface of the sample. The beam quality factor was measured by means of a High-Power-MicroSpotMonitor from Primes and was found to be M2 = 1.2. The beam diameter on the surface of the sample was varied by placing the sample off the focal plane. The experiments were carried out with laser powers $P_{L}$ ranging between 10.8 W and 1754 W, scanning speed v ranging between 0.7 m/s, 1 m/s, and 1.3 m/s, and beam diameters $d_{b}$ on the workpiece of 35 μm, 100 μm, and 200 μm (at 1/e^2^ of the maximum intensity in the center of the beam). The detailed experimental plan including the corresponding Péclet numbers and measurements, which are necessary for the determination of the process efficiency, the pore ratio, the aspect ratio, and the growth ratio, can be found in Appendix A, Table A1 and Table A2.

**Figure 3:**
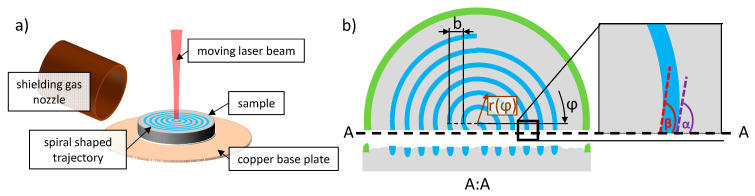
(**a**) Mounting of the samples in the experimental setup. The shielding gas nozzle was used to supply nitrogen to minimize oxidation. The laser beam was moved along a spiral-shaped trajectory by means of a scanner, (**b**) top view of a processed sample. A:A shows the plane of the cross-section. The detail shows the angles α and β that were considered for the evaluation when the cross-sections were not exactly centered to the origin of the spiral path.

The experiments were performed with the arrangement shown in Figure 3a. The samples were placed on a copper base plate, which enables a fast and precise exchange of the samples. In order to achieve a comparable process environment, a shielding gas nozzle was used to supply nitrogen to minimize oxidation during irradiation, as this is the industrial standard in TRUMPF [39] or EOS systems [40].

**Figure 4:**
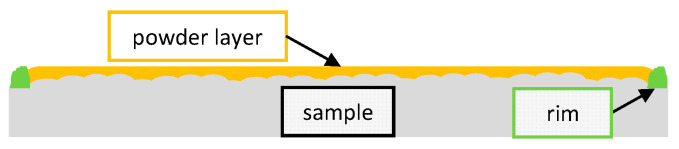
Illustration of the additively manufactured sample with the rim (green) to enable manual coating with reproducible height of the powder layers (orange).

In order to cover the pre-manufactured samples by a powder layer with a smooth surface corresponding to the ones of conventional processing, a rim was generated on the outer circumference of the samples as the final step of their production in the TruPrint3000 LPBF machine, see Figure 2. The height of the resulting rims of 82 randomly taken samples was measured using a confocal imaging profiler from Sensofar PLμ and was 80 μm with a standard deviation of 35 μm, which is in the range of the average diameter of the powder particles. The samples were put in a mount and an aluminum sheet with a thickness of *s* = 1.5 mm and a straight edge was applied as a scraper and pulled over the rim to manually coat the samples with powder, see Figure 4. The pre-manufactured rim on top of the sample ensures the repeatability of the manual coating process as it defines the height of the applied powder layer. The plain edge of the scraper was guided on the surface of the rim over the length of its thickness of *s* = 1.5 mm, which balances the height deviations of the rim and ensures constant layer heights.

After the application of the powder layer, the laser beam was moved along a spiral path with the radius $r\left(\varphi \right)=\frac{b}{2\cdot \pi }\cdot \varphi$ as a function of the angle of rotation φ with a distance of the lines of *b* = 0.5 mm as it is illustrated in Figure 3b. The starting point of the process was the center of the spiral. The spiral trajectory of the moving laser beam on these samples was also used to perform a calorimetric determination of the absorptance during LPBF, which was previously published in [37].

After laser processing, the cross-sections where produced by manually grinding and polishing the samples with a TegraPol-35 machine by Struers. Mechanical polishing was completed with a polishing suspension containing diamond particles with a diameter of 3 μm. Since the cross-sections were not always exactly centered to the origin of the spiral path, the measured cross-sectional areas and the width of the melt beads were multiplied by the factor sin((α+β)/2) given by the angles shown in Figure 3b.

The polished samples were anodically etched according to Barker [34] to be able to analyze the single melt beads. An Olympus BH2-UMA microscope with a Zeiss AxioCam MRc 5 camera was used to analyze the etched cross-sections with polarized illumination. Different magnifications between 50× and 500× were used, leading to a resolution of the recorded cross-sections ranging between 1.764 μm/pixel for the 50× magnification and 0.176 μm/pixel for the 500× magnification.

The material properties for AlSi10Mg, which were used for the calculation of the process efficiency (1), are shown in Table 1. The ambient temperature was set to 20 °C.

## 3. Results and Discussion

**Figure 5:**
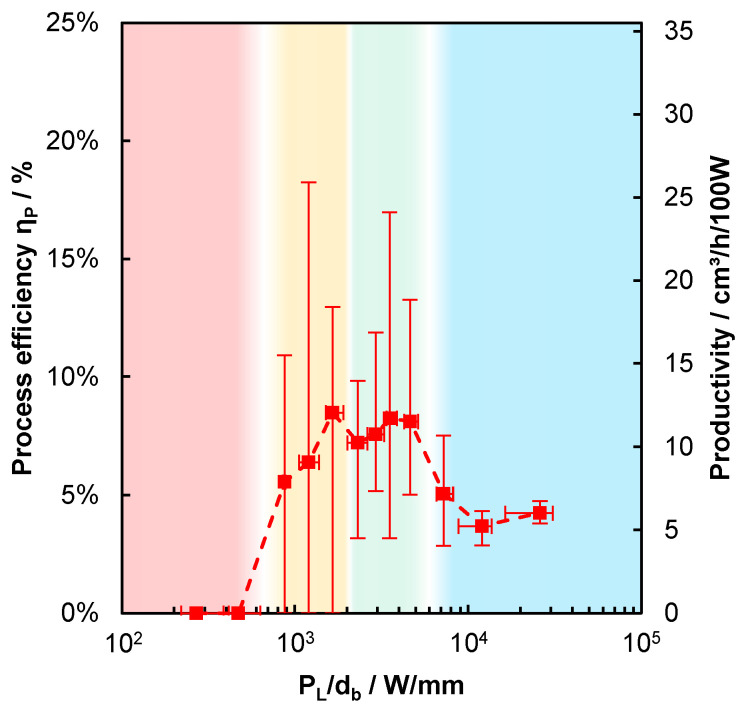
Experimentally determined process efficiency (1) and productivity (2) as a function of $P_{L}/d_{b}$. Red-colored area: complete failure of the additive process with $\eta_{P}=0$. Yellow-colored area: occasional failure of the additive process with minimum values of the error bars at 0% of $\eta_{P}$. Green-colored area: reproducible additive process with efficiencies always >0% in all experiments. Blue-colored area: reproducible additive process with average efficiencies <5%, but always >0%.

The process efficiency $\eta_{P}$ as determined using (1) for different sets of processing parameters is shown in Figure 5 as a function of $P_{L}/d_{b}$. As a further reference, the productivity can be calculated as follows:

(2) $${\dot{\Upsilon }}_{P}=\frac{A_{a}\cdot v}{P_{L}}.$$

This is proportional to $\eta_{P}$, and is given on the right-hand ordinate. A process efficiency of 1% here corresponds to a productivity of 1.42 cm^3^/h/100 W. As for all the following graphs, the shown values are the averaged results obtained with different $P_{L}$, $d_{b}$ and v. Each averaged result consists of the values of at least three parameter sets, where at least six cross-sections were analyzed in each individual parameter set. Thus, there are at least 18 analyzed cross-sections per averaged value. The parameter sets leading to the same or similar values of $P_{L}/d_{b}$ are summarized in one common averaged data point. The length of the error bars is given by the corresponding minimum and maximum of the averaged values. Four characteristic ranges can be identified, and are highlighted in Figure 5 by the red, yellow, green, and blue area. Up to $P_{L}/d_{b}$ = 610 W/mm, no material was added in general and the process efficiency equals zero, which is highlighted by the red area. For 870 W/mm ≲ $P_{L}/d_{b}$ ≲ 2000 W/mm, highlighted by the yellow area, material was added more frequently with increasing $P_{L}/d_{b}$, but the process still failed in some cases, as observed from the minimum values of the error bars at 0% of process efficiency. With $P_{L}/d_{b}$ exceeding a value of ≈2000 W/mm, which is highlighted by the green area, material is always reliably added during the processing and the lower end of the error bars is constantly >0%.

Between $P_{L}/d_{b}$ = 5200 W/mm and $P_{L}/d_{b}$ = 7200 W/mm, the process efficiency was found to decrease from 8% to 5%, and a further increase of $P_{L}/d_{b}$ did not lead to process efficiencies exceeding 5%, which is highlighted by the blue area in Figure 5. In view of the process efficiency, this suggests that the LPBF process should be performed within the range of approximately 2000 W/mm ≲ $P_{L}/d_{b}$ ≲ 5200 W/mm, to achieve the highest process efficiency.

**Figure 6:**
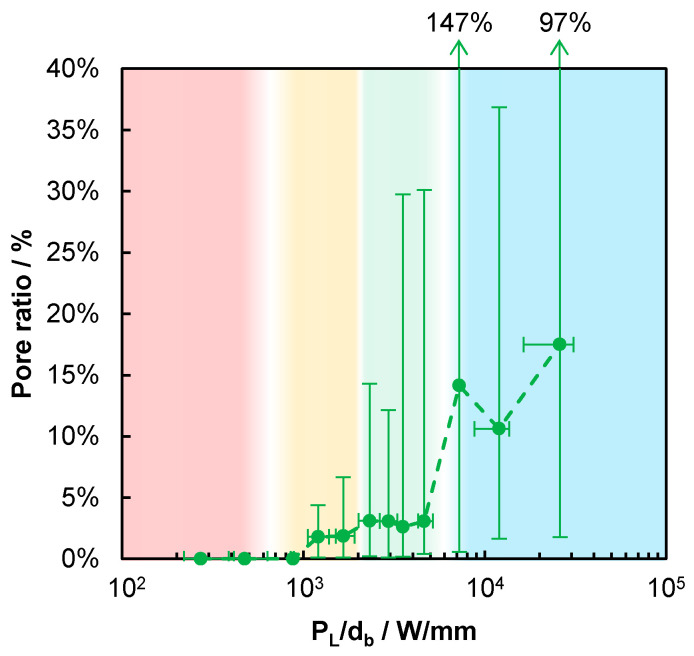
Pore ratio $A_{P}/A_{a}$ as a function of $P_{L}/d_{b}$. The highlighted areas correspond to the highlighted areas in Figure 5 (Red-colored area: complete failure of the additive process with $\eta_{P}=0$. Yellow-colored area: occasional failure of the additive process with minimum values of the error bars at 0% of $\eta_{P}$. Green-colored area: reproducible additive process with efficiencies always >0% in all experiments. Blue-colored area: reproducible additive process with average efficiencies <5%, but always >0%).

The relative porosity $A_{P}/A_{a}$ of the produced beads is shown in Figure 6. The highlighted areas in Figure 6 correspond to the highlighted areas in Figure 6. In the range up to $P_{L}/d_{b}$ = 610 W/mm, the averaged pore ratio is 0%, since no material is added, see Figure 5. In the range 870 W/mm ≲ $P_{L}/d_{b}$ ≲ 2000 W/mm, highlighted by the yellow area, for $P_{L}/d_{b}$ ≲ 1000 W/mm the averaged pore ratio is 0%, whereas in the range 1000 W/mm ≲ $P_{L}/d_{b}$ ≲ 2000 W/mm, the averaged pore ratio is less than 2% and the maximum given by the upper end of the error bars never exceeds 7%. Due to the failed processes with process efficiencies of 0%, as shown in Figure 5 and highlighted by the same red and yellow areas in Figure 6, the processes up to $P_{L}/d_{b}$ = 2000 W/mm result in a lack of fusion porosity when a hatch would be applied. The detection of a lack of fusion porosity was not possible within the single bead analysis. In the range 2000 W/mm ≲ $P_{L}/d_{b}$ ≲ 5200 W/mm, the averaged porosity is slightly elevated, though still below 3.5%, whereas the variation grows with increasing $P_{L}/d_{b}$, as observed from the upper end of the error bars, which is highlighted by the green area. For $P_{L}/d_{b}$ ≳ 5200 W/mm, the average porosity exceeds 10% and the variation in the porosity (given by the error bars) is further increased, which is highlighted by the blue area. The $P_{L}/d_{b}$ ranges highlighted in green and blue in Figure 6 are the same as the green and blue ones in Figure 5. Together with the findings on the process efficiency, this suggests that the optimum process regime is found in the range 2000 W/mm ≲ $P_{L}/d_{b}$ ≲ 5200 W/mm.

**Figure 7:**
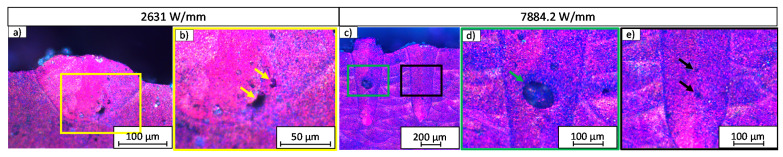
Exemplary images of melt beads with pores. (**a**) Melt bead generated with $P_{L}/d_{b}=$ 2631 W/mm with irregular pores. (**b**) Detail of (**a**) with irregular pores highlighted with yellow arrows. (**c**) Melt beads generated with $P_{L}/d_{b}$ = 7884.2 W/mm with one great spherical pore inside the melt bead (left). Same parameter set on the melt bead on the right side with the formation of small spherical pores only. (**d**) Detail of (**c**) (left) with one great spherical pore highlighted with green arrow. (**e**) Detail of (**c**) (right) with small spherical pores highlighted with black arrows.

Inside the melt beads, different types of pores can be distinguished. In Figure 7, the melt beads generated with different process parameters are shown. In Figure 7a, a melt bead is shown, which was generated with $P_{L}/d_{b}$ = 2631 W/mm. Small and irregular pores are present inside the melt bead, as highlighted in Figure 7b, with the yellow arrows. In Figure 7c, two melt beads are shown, which were both generated with $P_{L}/d_{b}$ = 7884.2 W/mm. Inside the left melt bead, one great spherical pore can be detected, which is highlighted in Figure 7d, with the green arrow, whereas inside the right melt bead, only very small spherical pores are present, which is highlighted in Figure 7e, with the black arrows. This variation in the pores leads to the high range of minimum and maximum values of the pore ratio, as shown in Figure 6.

In order to further analyze the influence of the value of $P_{L}/d_{b}$ on the characteristics of the process, the growth ratio given by $A_{a}/A_{tot}$ and the aspect ratio depth/width of the beads, see Figure 1, were analyzed, as shown for five examples in Table 2. Up to the example (c), the process is found to be in the mode of heat conduction melting (HCM), since the depth of the melt bead is smaller than its width. In contrast, the shape of the bead with an aspect ratio of >>1 of the example (e) clearly indicates that a keyhole must have been formed, which corresponds to the mode of deep-penetration melting (DPM). The aspect ratio of the bead in example (d) is 0.9, which means that the process is running in the transition between HCM and DPM, where evaporation is thought to already occur, but is not intense enough to form a deep keyhole. This transitional range, with an aspect ratio of 1 ± 0.1, is referred to as the mode of key-bowl melting (KBM) in the following.

**Figure 8:**
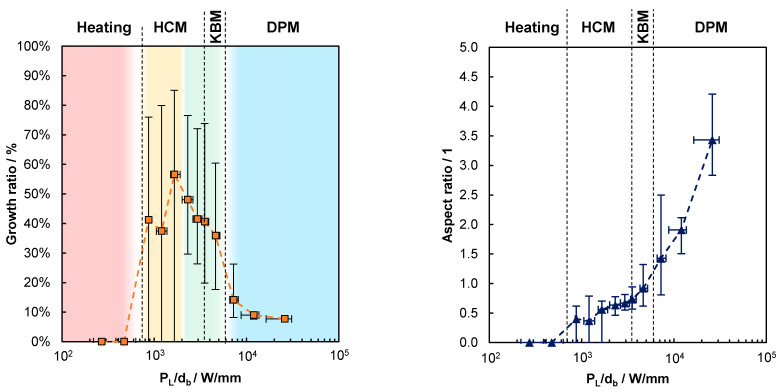
(**Left**) growth ratio $A_{a}/A_{tot}$ as function of $P_{L}/d_{b}$ with the corresponding processing modes. Red-colored area: complete failure of the additive process with $\eta_{P}=0$. Yellow-colored area: occasional failure of the additive process with minimum values of the error bars at 0% of $\eta_{P}$. Green-colored area: reproducible additive process with efficiencies always >0% in all experiments. Blue-colored area: reproducible additive process with average efficiencies <5%, but always >0%. (**Right**) aspect ratio depth/width of the bead as a function of $P_{L}/d_{b}$ with the corresponding processing modes.

The decreasing growth ratio with increasing depth of the melt bead observed with increasing $P_{L}/d_{b}$ is consistent with the decreased process efficiency observed in the blue-colored area of Figure 5. In fact, the dependence of the growth ratio on $P_{L}/d_{b}$, shown by the left chart in Figure 8, qualitatively corresponds to the one of the process efficiency in Figure 5, which is highlighted by the same-colored areas. Again, the growth ratio equals zero for $P_{L}/d_{b}$ ≲ 610 W/mm, which means that the material is only heated, but not melted—referred to as the heating mode. For 870 W/mm ≲ $P_{L}/d_{b}$ ≲ 2000 W/mm, the average growth ratio is >0, but with large variations, as observed from the error bars that still reach down to zero at the lower end. As from $P_{L}/d_{b}$ = 2000 W/mm, a further increase in $P_{L}/d_{b}$ leads to a continuous decrease in the growth ratio, with a significant step between 5200 W/mm and 7200 W/mm, where a deep keyhole is formed, as can be observed from the right graph in Figure 8.

The growth ratio is strongly related to the aspect ratio, which is shown on the right-hand side in Figure 8. Up to $P_{L}/d_{b}$ = 610 W/mm, no melting occurred, and thus, the powder and substrate were heated to less than the melting temperature (heating mode). Melting, but no evaporation of the material, occurs in the mode of heat conduction melting (HCM), in the range of 870 W/mm ≲ $P_{L}/d_{b}$ ≲ 3500 W/mm, where the aspect ratio is still below 0.7. In the range of 3500 W/mm ≲ $P_{L}/d_{b}$ ≲ 6000 W/mm, the aspect ratio of the melt bead is around one, which is an indication of shallow keyholes. In this mode, referred to as key-bowl melting (KBM), both the growth ratio and the process efficiency exhibit high values. When $P_{L}/d_{b}$ exceeds a value of ≈6000 W/mm, the process runs in the mode of deep-penetration melting (DPM), with average aspect ratios exceeding 1.4. This leads to excessive melting of the layers below which results in a distinct decrease in both the growth ratio and the process efficiency, compare to Figure 8(left) and Figure 5.

**Figure 9:**
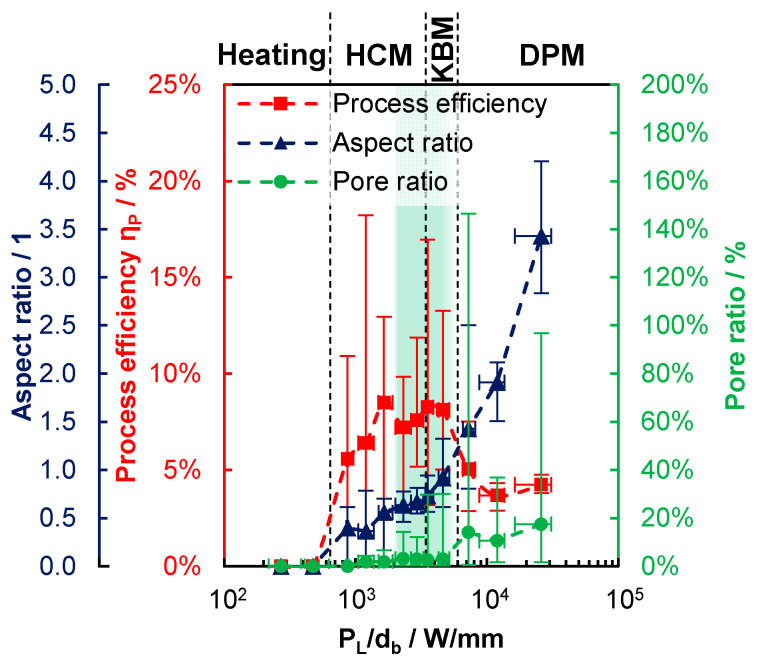
Process efficiency (left, red), aspect ratio (left, blue) and pore ratio (right, green) as functions of $P_{L}/d_{b}$. Green-colored area: process window for highly efficient laser-based powder bed fusion of AlSi10Mg with reduced pore formation.

All these findings are superimposed in Figure 9, which helps to identify the most suitable process window. Again, one sees that for 2000 W/mm ≲ $P_{L}/d_{b}$ ≲ 5200 W/mm, where continuous beads are reliably formed, the process efficiency is maximized, while the pore ratio is low, which leads to high-quality parts, which is highlighted by the same green range that was already identified in Figure 5, Figure 6 and Figure 7. In this range, the combination of $P_{L}$ and $d_{b}$ leads to sufficient heating, in which the applied powder layer and the substrate below can generate a continuous melt bead. The amount of the required energy between the applied powder layer and the substrate is balanced, as shown by the growth ratio, which leads to the highest achievable process efficiencies. Due to the comparatively steady melting process, the gaps between the powder particles can outgas, and beads with low pore ratios are formed. In the KBM process for 3500 W/mm ≲ $P_{L}/d_{b}$ ≲ 5200 W/mm, there is no fluctuation of the shallow keyholes, so no keyhole-induced pores are formed.

When $P_{L}/d_{b}$ falls below a value of ≈2000 W/mm, the heating is no longer sufficient to form a sufficiently large melt pool. This results in process failures, no continuous beads are produced, and the minimum process efficiency drops to 0%. The application of these parameters in an LPBF machine leads to the well-known lack of fusion error patterns.

When $P_{L}/d_{b}$ falls below a value of ≈610 W/mm, the heating is no longer sufficient to generate melt pools at all. The sample is only heated up and balling may be formed.

When $P_{L}/d_{b}$ exceeds a value of ≈5200 W/mm, the pore ratio increases and the process efficiency drops, which can both be attributed to the deep and narrow keyhole of the DPM, as confirmed by the increased aspect ratio of the beads. Here, the evaporation temperature is far exceeded and the vapor pressure forms the deep keyhole. Due to the formation of the deep keyhole, deeper layers are melted, which lowers the process efficiency. Furthermore, since this keyhole fluctuates greatly, occasional keyhole break-offs occur, resulting in the large spherical pores of entrapped gas.

In the overall result, the process modes and their derivation via $P_{L}/d_{b}$ show a clear predictive ability about the LPBF process of AlSi10Mg, and its parameter limits as a function of the incident laser power $P_{L}$ and the beam diameter $d_{b}$ on the surface of the workpiece.

## 4. Conclusions

Previous studies have shown the influence of the hatch distance and height of the powder layer on the process efficiency [4]. In the present study, we have investigated the influence of the incident laser power $P_{L}$ and the beam diameter $d_{b}$ on the surface of the workpiece, and their influence on the process efficiency, using the single bead on additively pre-manufactured samples. The knowledge of the process efficiency defined by the incident laser power $P_{L}$ and the beam diameter $d_{b}$ on the surface of the workpiece is necessary to improve the overall efficiency, considering the other parameters (i.e., hatch distance, layer height, hatch strategy, temperature of the substrate) used for LPBF.

It was shown that the $P_{L}/d_{b}$ is a suitable quantity to describe the process modes (heating, HCM, KBM, and DPM) in LPBF, below the Péclet number of 16/π, as it has already been shown for laser hardening, laser heat-conduction welding, and laser deep-penetration welding [30,31]. With this quantity, and based on the presented results, it is possible to distinguish the four process modes that can be achieved in LPBF. Heating and the initiation of HCM lead to failed processes, resulting in balling and a lack of fusion, which can be observed at $P_{L}/d_{b}$ < 2000 W/mm. The formation of a deep keyhole at DPM at $P_{L}/d_{b}$ > 5200 W/mm leads to the re-melting of many previously added and deeper layers, which is associated with a decrease in the process efficiency and a decrease in the growth ratio. In addition, the presence of the deep keyhole leads to an increased pore ratio.

The determined process window was achieved at a height of the powder layer of 80 μm, with a standard deviation of 35 μm. According to the results presented by Ye et al. [36], the height of the powder layer influences the absorptance of the laser beam during the LPBF process in the process mode of heating and HCM. Here, a transition of the process window is assumed to apply with the variation in the height of the powder layer. As soon as the keyhole was formed, no influence of the height of the powder layer was present, and the transition between HCM and DPM was not affected by the height of the powder layer. This leads to the assumption that the magnitude of the process efficiency at HCM is correlated with the absorptance, and thus with the height of the powder layer, while the principal behavior of the analyzed quantities (i.e., process efficiency, pore ratio, growth ratio, and aspect ratio), and the transition between HCM, KBM, and DPM, as a function of $P/d_{b}$, should not be affected. This has to be analyzed in further studies.

Furthermore, $P/d_{b}$ below the Péclet number of 16/π enables process-oriented adjustment of the process parameters directly in the laboratory. This makes it easy to quickly set the first process parameters for a good process window, as the beneficial process modes are shown as a function of $P/d_{b}$. This means that enlarged diameters of the laser beam are directly coupled to a laser power at which a known process takes place, and vice versa. Nevertheless, for more detailed process windows, a further parameter study has to be performed.

In summary, the optimum process window for the single-bead LPBF of AlSi10Mg, yielding both a high process efficiency and reduced pore formation, was found at 2000 W/mm ≲ $P_{L}/d_{b}$ ≲ 5200 W/mm, in which the process changes from the HCM to KBM at about $P_{L}/d_{b}$ ≈ 3500 W/mm. Further research will be devoted to the influence of the parameters used on the mechanical properties of the additively manufactured parts.

## Figures and Tables

**Table 1:** Material properties used for AlSi10Mg [41,42].

$c_{p}$ in $\frac{J}{kg\cdot K}$	$T_{m}$ in °C	$h_{s}$ in $\frac{kJ}{kg}$	ρ in $\frac{kg}{m^{3}}$	$\lambda_{th}$ in $\frac{W}{m\cdot K}$
910	585	410	2680	150

**Table 2:** Influence of $P_{L}/d_{b}$ on the resulting growth ratios and aspect ratios. Parameters: $d_{b}$ = 100 μm, v = 1.3 m/s.

**Corresponding Image**	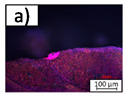	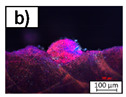	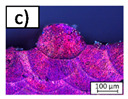	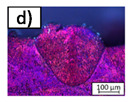	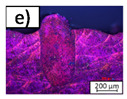
$P_{L}/d_{b}$	1676 W/mm	2929 W/mm	3523 W/mm	6989 W/mm	13,627 W/mm
**Growth ratio** $A_{a}/A_{tot}$	69.3%	58%	48.8%	26.2%	7.7%
**Aspect ratio** ***depth/width***	0.65	0.71	0.67	0.90	2.10

## Data Availability

Data are contained within the article.

## Appendix A

**Table A1:** Experimental plan and detailed results of the cross-sectional areas of the investigation. Each parameter set was repeated three times and at least six melt beads were analyzed per parameter set.

*d_b_* in μm	*v* in m/s	*P_L_* in W	Cross-Sectional Area of the Melt Bead *A_tot_* in μm^2^			Cross-Sectional Area of the Added Material *A_a_* in μm^2^			Pore Area Inside the Melt Bead *A_p_* in μm^2^		
			avg	−	+	avg	−	+	avg	−	+
35	0.7	10.8	N/A	N/A	N/A	N/A	N/A	N/A	N/A	N/A	N/A
		57.1	2986.3	2386.3	9724.3	2771.7	2555.6	10,824.9	72.9	0	0
		87.9	4360.6	3259.4	5487.9	2014.2	2014.2	3852.5	228.3	59.7	59.7
		118.9	13,515.2	10,778.5	6390.6	4072	4072	7358.7	55.9	42.3	56.7
		149.8	18,592	4594	5826.3	15,575	15,575	86,535.5	208	148	452.1
		180.7	26,601.9	4419.4	4369.3	6127	6127	5128.3	175.8	74	48.9
		1078	394,554	60,312.3	54,651.3	29,120	15,737	14,748.1	4737.3	4227	18,794
	1	48.3	N/A	N/A	N/A	N/A	N/A	N/A	N/A	N/A	N/A
		70.3	3371.8	3243.7	6909.3	4084.1	3914	5828.4	95.3	79.4	52
		92.4	5068.2	3946.3	4419.2	4711.3	4692.2	3797.4	98.6	72.3	57.5
		114.4	6455.1	5404.3	6287.2	4437.8	4253.5	8582.7	393.8	0	0
		136.5	11,876	5274.7	4968	5332.7	4451.9	6278.1	161.1	77.8	213.2
		158.7	16,407.2	6510.5	5267.3	6952	4521.7	6868.2	286.9	204.7	309.8
		180.7	19,727.7	7524.7	6490	5456.1	2934.8	4876.3	232.1	186.2	247.1
	1.3	13.5	N/A	N/A	N/A	N/A	N/A	N/A	N/A	N/A	N/A
		43.9	N/A	N/A	N/A	N/A	N/A	N/A	N/A	N/A	N/A
		57.1	N/A	N/A	N/A	N/A	N/A	N/A	N/A	N/A	N/A
		87.9	3657.6	3157.6	5467.6	2735	2580.8	5724.3	15.9	0	0
		118.9	2495.5	2350.4	2821.4	1354.5	1306.1	2514.1	17.7	15.3	18.4
		149.8	9888.2	1926.1	4574.1	6921.6	5496.3	12,076.9	212.4	61.7	92.1
		180.7	11,299.3	6016.4	3564.5	6975	6591.5	4593.1	49.9	22.6	34
		1078	180,400	23,820	23,068.8	16,237	9070.8	27,598.7	4697.5	3835	11,011
100	0.7	43.9	N/A	N/A	N/A	N/A	N/A	N/A	N/A	N/A	N/A
		105.6	10,222.6	9143.3	14,254.9	8416.5	8416.5	14,820.7	50	42.7	51.8
		167.6	14,416.6	8736.3	11,531.5	10,619	9202.4	14,568.5	123.8	10.8	13.3
		228.2	13,804.1	5865.7	8237.1	4809.2	3576.9	5773	188.3	120.3	186.9
		292.9	26,456.1	15,295.4	9902	9336.9	6394.5	10,465.4	260.6	79.2	105.4
		352.3	45,789.7	8098.3	11,834.4	39,395	25,877	150,726	310.4	163.3	325.4
		698.9	138,837	17,793	14,695.7	29,148	11,730	22,574.4	3336.3	2010	2817.3
		1363	357,209	66,947.2	52,143	42,018	19,432	27,513.7	8367.8	6808	7119
		1632	426,680	56,238.2	105,876	29,120	15,737	14,748.1	2574.2	858.3	611.6
	1	41.2	N/A	N/A	N/A	N/A	N/A	N/A	N/A	N/A	N/A
		63.3	N/A	N/A	N/A	N/A	N/A	N/A	N/A	N/A	N/A
		85.1	1943.5	1427.6	2160.2	529	529	1587	N/A	N/A	N/A
		107	1773.6	1738.2	3495.8	1879.3	1563.2	2562.7	81.9	0	0
		128.9	8852.1	7674.6	9953.4	7112.6	7112.6	7495.2	105	63.6	63.6
		150.8	9946.5	9026.1	5845.2	8702.2	8043	10,820.4	99.4	90.6	82.4
		191.2	9337.3	4400.5	3945.2	5970.4	4204.9	2584.4	219.5	184.2	177.9
		263.1	26,178.3	6434.4	8174.3	13,790	8417.5	9140.5	219.2	141.3	133.4
		307	35,349.2	14,604.2	12,119.2	14,822	10,375	11,245.5	462.7	446.5	1337.4
		350.8	45,550.7	45,097.2	38,301.8	10,472	10,472	14,050.3	698.5	609.2	2418.2
		438.5	67,637.7	12,136.8	14,550.1	13,077	8823.7	14,668.9	877.8	688.8	3060.3
		657.8	113,805	26,788.1	22,695.1	12,060	11,286	18,066.9	1426.7	1105	3237
		788.4	152,694	36,831.1	44,772.8	19,731	19,731	25,206.4	2794.4	2687	6906.3
		1316	230,919	107,119	146,329	30,949	30,949	119,632	15,056	12,600	27,450
	1.3	43.9	N/A	N/A	N/A	N/A	N/A	N/A	N/A	N/A	N/A
		105.6	N/A	N/A	N/A	N/A	N/A	N/A	N/A	N/A	N/A
		167.6	8069.2	7006.8	7445.9	6855.5	6462	9754.2	45	0	0
		228.2	10,912.9	2660.2	3216.5	5898.5	2875.6	1188.4	110.2	33.7	54.6
		292.9	18,373.8	10,166.8	19,173.7	10,641	9546.6	22,828.9	76	48.5	35.2
		352.3	22,394.3	10,133.2	8331.2	12,061	8877.2	11,105.8	63	0	0
		698.9	60,945.6	16,900.7	8415.1	18,309	10,050	10,857	852.4	0	0
		1363	155,761	18,977.9	25,489	16,574	14,712	18,713.2	1286.5	1018	904.4
200	0.7	43.9	N/A	N/A	N/A	N/A	N/A	N/A	N/A	N/A	N/A
		176.4	14,325.7	12,556	11,009.4	11,071	10,194	9206.4	209.5	168.4	171.8
		310	25,673.7	11,811	26,850.3	16,096	15,562	34,363.1	456	52.7	41.9
		439.7	36,922.1	13,300.9	15,975.2	16,854	11,725	12,943.7	517.1	478.1	977
		567.8	52,721.1	19,365.4	21,517.8	17,113	10,508	17,117.9	497	289.7	396.3
		698.9	71,496.9	27,106	20,464.6	18,126	10,099	16,031.8	1027.2	784	1999.8
		869.4	107,717	14,561.1	21,598.6	31,041	12,651	13,346.5	561	106.6	165.3
		1632	337,112	53,493.1	108,988	53,807	27,673	43,281	3012.7	1692	2944.8
	1	43.9	N/A	N/A	N/A	N/A	N/A	N/A	N/A	N/A	N/A
		87.7	N/A	N/A	N/A	N/A	N/A	N/A	N/A	N/A	N/A
		175.4	10,882.4	10,237	27,552.9	8403.9	7911.6	27,943	56.3	13.8	13.8
		263.1	24,372.5	23,224.3	32,500.1	19,571	18,959	35,598.6	146.7	124.6	105.2
		350.8	28,002.3	11,134.8	9602	12,829	12,330	14,220.7	116.9	87.9	279.8
		438.5	37,578.8	13,561.5	15,953.1	17,500	9785.3	10,491.4	57.8	22.2	30.4
		526.2	44,453.9	16,253.8	13,343.4	14,465	7233.9	8611.7	516.6	449.6	1043.3
		657.8	62,643.4	18,639.3	26,984.1	19,702	6519.6	16,267.1	399	332.8	370.7
		1316	171,002	33,319.2	82,957.2	46,390	30,134	36,824.5	4057.6	3798	10,391
		1754	266,381	41,890.5	20,669.7	28,720	13,913	40,968.8	1203.9	736.7	807.9
	1.3	43.9	N/A	N/A	N/A	N/A	N/A	N/A	N/A	N/A	N/A
		176.4	N/A	N/A	N/A	N/A	N/A	N/A	N/A	N/A	N/A
		310	17,392.2	14,826.3	15,837.5	13,252	11,963	17,503.3	235	152.3	203.9
		439.7	21,598.9	6957.5	18,684.6	10,862	9847.9	21,240.9	320.5	95.7	95.7
		567.8	26,891.1	23,175.6	9934.9	14,008	10,574	14,564.1	208.3	143.5	156.6
		698.9	50,002.9	8281.3	14,579.8	29,866	12,860	17,493.9	472.7	0	0
		869.4	33,793.6	12,451.3	20,467.7	17,677	17,677	22,919.4	495.5	372.6	453.8
		1635	131,138	34,559.2	24,945.7	35,506	24,488	34,417.5	3588.4	2876	8015

**Table A2:** Experimental plan and detailed results of the Péclet numbers, the cross-sectional depths, and widths of the melt beads of the investigation. Each parameter set was repeated three times and at least six melt beads were analyzed per parameter set.

*d_b_* in μm	*v* in m/s	*P_L_* in W	Péclet-Number *Pe* in 1	Depth of the Melt Bead in μm			Width of the Melt Bead in μm		
				avg	−	+	avg	−	+
35	0.7	10.8	0.4	N/A	N/A	N/A	N/A	N/A	N/A
		57.1	0.4	39.3	14.3	116.2	69.3	44.4	123
		87.9	0.4	53.3	27.4	99	101	60.6	147.2
		118.9	0.4	97.5	34.7	146.2	148	69.4	198.8
		149.8	0.4	125	71.3	203	170	143	222.3
		180.7	0.4	161	92	261.1	218	184	387.3
		1078	0.4	825	177	1540	440	353	526.7
	1	48.3	0.57	N/A	N/A	N/A	N/A	N/A	N/A
		70.3	0.57	43.6	6.9	126.3	72	33.8	110.8
		92.4	0.57	57.6	26.6	108.5	87.8	59.4	121.9
		114.4	0.57	65.8	23.6	129.8	114	81.3	146.2
		136.5	0.57	96.3	58.7	153.3	138	117	166
		158.7	0.57	116	66.2	181.7	162	132	183.5
		180.7	0.57	131	74.2	227.3	184	148	323.9
	1.3	13.5	0.74	N/A	N/A	N/A	N/A	N/A	N/A
		43.9	0.74	N/A	N/A	N/A	N/A	N/A	N/A
		57.1	0.74	N/A	N/A	N/A	N/A	N/A	N/A
		87.9	0.74	45.5	10.1	124.8	71.7	37.2	112.9
		118.9	0.74	34.3	8.5	70.9	64.4	24.4	100.9
		149.8	0.74	89.6	42.9	189.4	122	85.8	143.5
		180.7	0.74	92.2	51.2	150.3	136	103	167
		1078	0.74	555	102	1105	236	205	262.7
100	0.7	43.9	1.14	N/A	N/A	N/A	N/A	N/A	N/A
		105.6	1.14	70.5	22.1	149.9	116	60.9	191
		167.6	1.14	96	61.6	185.4	170	136	228.5
		228.2	1.14	95.3	49	134.7	198	169	240.4
		292.9	1.14	138	77.1	191.7	255	196	305.5
		352.3	1.14	184	139	233.2	314	279	354.2
		698.9	1.14	343	197	528.3	426	393	474.4
		1363	1.14	653	181	1147	500	362	632.9
		1632	1.14	761	195	1578	457	390	503.9
	1	41.2	1.63	N/A	N/A	N/A	N/A	N/A	N/A
		63.3	1.63	N/A	N/A	N/A	N/A	N/A	N/A
		85.1	1.63	33	11.4	52.1	74.6	44.9	99.3
		107	1.63	30.9	4.2	68.6	64.5	9.8	135.8
		128.9	1.63	73.2	27.2	154	132	57.1	236.8
		150.8	1.63	77.9	24	134.4	137	48.1	229.4
		191.2	1.63	79.6	50.3	129.6	147	101	192
		263.1	1.63	138	102	222.5	229	204	253.5
		307	1.63	164	104	301.2	256	208	323.2
		350.8	1.63	180	13	348.8	250	26	449.2
		438.5	1.63	256	117	428.7	281	233	308.7
		657.8	1.63	348	119	719.6	293	238	340.4
		788.4	1.63	433	126	929.9	289	251	337.8
		1316	1.63	537	99.1	1302	251	198	335.1
	1.3	43.9	2.11	N/A	N/A	N/A	N/A	N/A	N/A
		105.6	2.11	N/A	N/A	N/A	N/A	N/A	N/A
		167.6	2.11	70	22	138	122	74.5	169.7
		228.2	2.11	88.3	55.5	148	139	111	166.6
		292.9	2.11	110	73.6	208.8	182	159	240.8
		352.3	2.11	125	82.7	177.7	214	165	233.9
		698.9	2.11	217	135	361.5	309	269	359.4
		1363	2.11	376	129	696.9	289	259	311.9
200	0.7	43.9	2.28	N/A	N/A	N/A	N/A	N/A	N/A
		176.4	2.28	95.3	35.6	147.5	174	78.6	254.9
		310	2.28	130	72.7	240.8	245	205	283
		439.7	2.28	160	106	225.2	314	289	330.4
		567.8	2.28	195	139	230.9	368	328	424.2
		698.9	2.28	222	176	290.5	411	356	485.6
		869.4	2.28	274	221	381.2	462	441	491.7
		1632	2.28	513	340	679.6	786	680	1105
	1	43.9	3.25	N/A	N/A	N/A	N/A	N/A	N/A
		87.7	3.25	N/A	N/A	N/A	N/A	N/A	N/A
		175.4	3.25	77.6	15.5	219.3	139	53.1	219.8
		263.1	3.25	126	26.1	304.5	195	73.5	254.8
		350.8	3.25	138	99.3	209.2	244	208	293.7
		438.5	3.25	164	128	253.7	282	255	326.7
		526.2	3.25	178	126	243.9	313	251	365.2
		657.8	3.25	215	122	287.6	354	245	474.2
		1316	3.25	369	166	650.2	439	333	630.3
		1754	3.25	481	185	892	479	369	570.3
	1.3	43.9	4.23	N/A	N/A	N/A	N/A	N/A	N/A
		176.4	4.23	N/A	N/A	N/A	N/A	N/A	N/A
		310	4.23	106	38.6	211.1	177	89.1	228.1
		439.7	4.23	123	102	192.9	235	203	285.7
		567.8	4.23	136	41.4	177.8	259	82.7	303
		698.9	4.23	195	147	305.9	324	293	385.2
		869.4	4.23	160	120	233.8	286	241	335.8
		1635	4.23	316	197	521.5	450	394	522.7
